# Unusual presentation of an usual condition—cystic ganglionosis

**DOI:** 10.1259/bjrcr.20150231

**Published:** 2015-09-10

**Authors:** Anuradha Rao, Srivalli Nandikoor, Govindarajan Mallarajapatna, Prabhu Meghanathan

**Affiliations:** ^1^ Department of Radiology, Apollo Hospitals, Bangalore, India; ^2^ Department of Pathology, Apollo Hospitals, Bangalore, India

## Abstract

Cystic ganglionosis is an unusual benign condition, which presents as multiple ganglion cysts involving multiple joints. The case we report here is probably the first case of an adult reported in the literature. The only other case of multiple ganglion cysts reported in the literature is that of an 11-year-old child with ganglion cysts in multiple joints. A 57-year-old female presented with an approximately 4-year history of pain and swelling below the left knee and in the right hand. An MRI of the left knee showed multiloculated cystic-intensity lesions around the knee joint with intra-/extra-articular and intraosseous components. Similar lesions were noted around the right knee joint, right wrist and ring finger. With all the above findings and in view of multiple joint involvement, the possibility of cystic ganglionosis or multiple giant ganglion cysts was considered and confirmed by biopsy of one of the knee lesions. It is because of this rarity and the unusual presentation involving multiple joints that we have reported this case. The importance of the diagnosis is that unnecessary surgical intervention and hospitalization can be avoided, provided the patient’s symptoms are not severe.

## Summary

Ganglion cysts are common lesions presented by the patients. Ganglia are not true tumours. Ganglia arise from the joint capsules, bursae, ligaments, tendons and subchondral bone. Hands, wrists and feet are the sites where they are commonly seen. They are usually asymptomatic; however, the mass effect on the adjacent tissue can sometimes be the clinical presentation.^1^ Here we discuss a rare case of multiple ganglion cysts presenting predominantly with pain and swelling of the knee that we diagnosed as “cystic ganglionosis”. To our knowledge, no case of multiple ganglion cysts in adults has been reported in the medical literature. Only one case of multiple ganglion cysts has been reported in an 11-year-old child.^2^ We have reported this case because of this rare multiplicity, the unusual presentation involving the bilateral knees and Hoffa’s fat pad, together with multiple intraosseous lesions in a single patient. This is probably the first case of an adult reported in the literature.

## Case report

**Figure 1.**
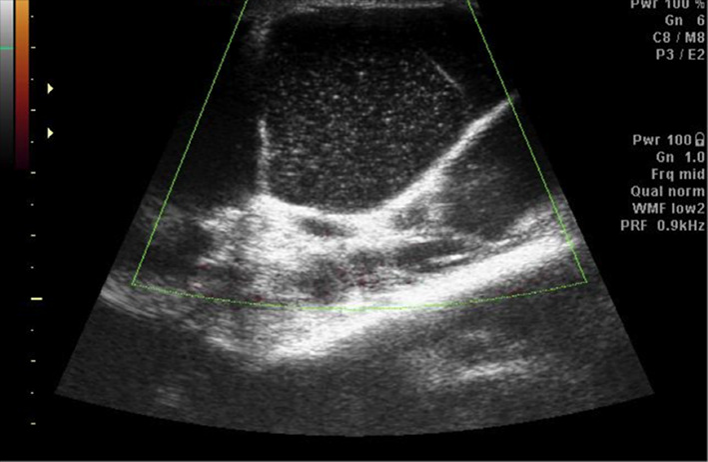
Ultrasound image of the knee showing multiple loculated cystic lesions involving the subcutaneous planes and the popliteal fossa with diffuse internal echoes and septations. No internal vascularity is seen.

**Figure 2.**
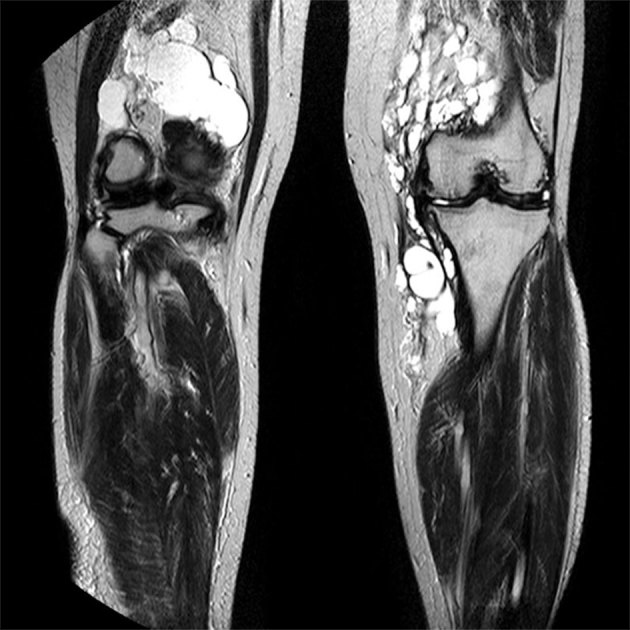
Coronal *T*_2_ MRI of bilateral knees showing multiloculated cystic lesions with intra- and extra-articular components.

**Figure 3.**
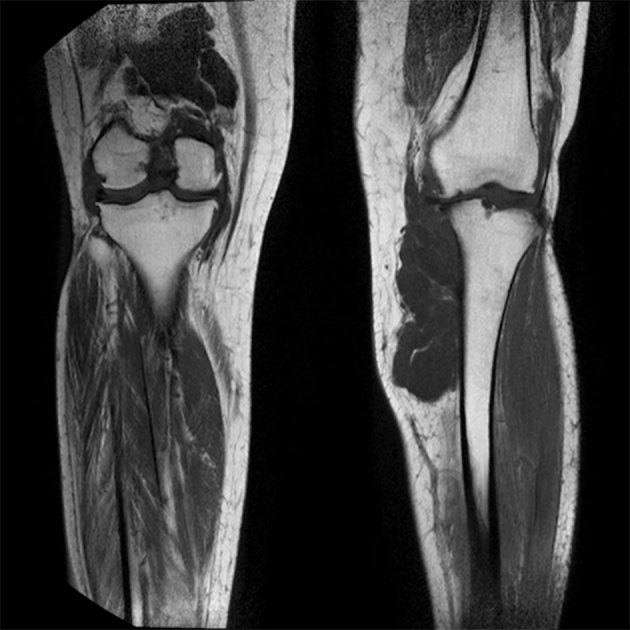
Coronal *T*_1_ MRI of bilateral knees showing multiloculated hypointense (cystic) lesions with intra- and extra-articular components.

**Figure 4.**
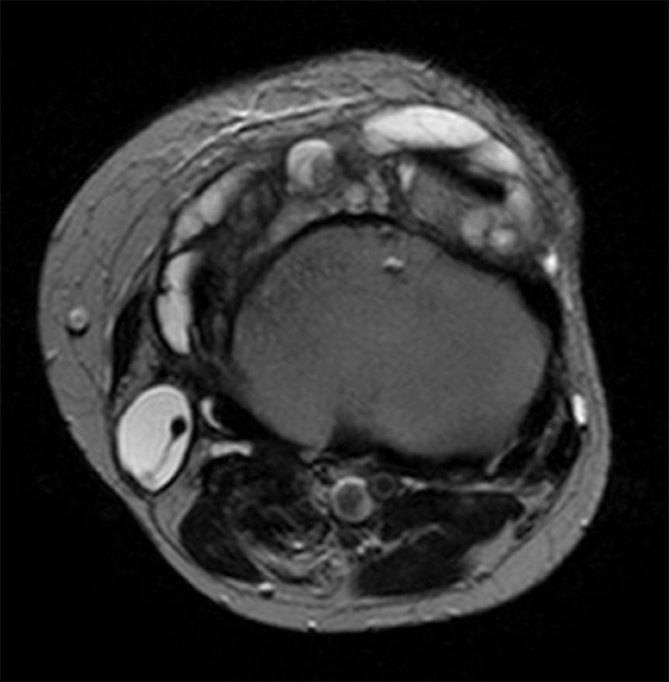
Axial *T*_2_ MRI of bilateral knees showing hyperintense lesions insinuating between the tendons and muscles, and also demonstrating an intra-articular component.

**Figure 5.**
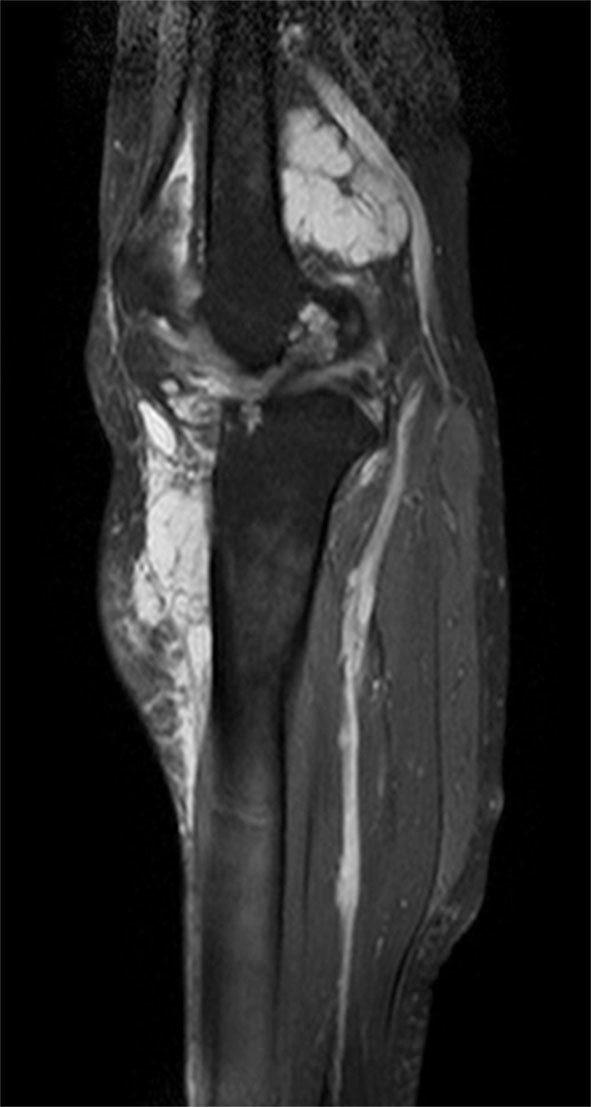
Sagittal short tau inversion-recovery MRI of the knee showing involvement of Hoffa’s fat pad and the femoral intraosseous component.

**Figure 6.**
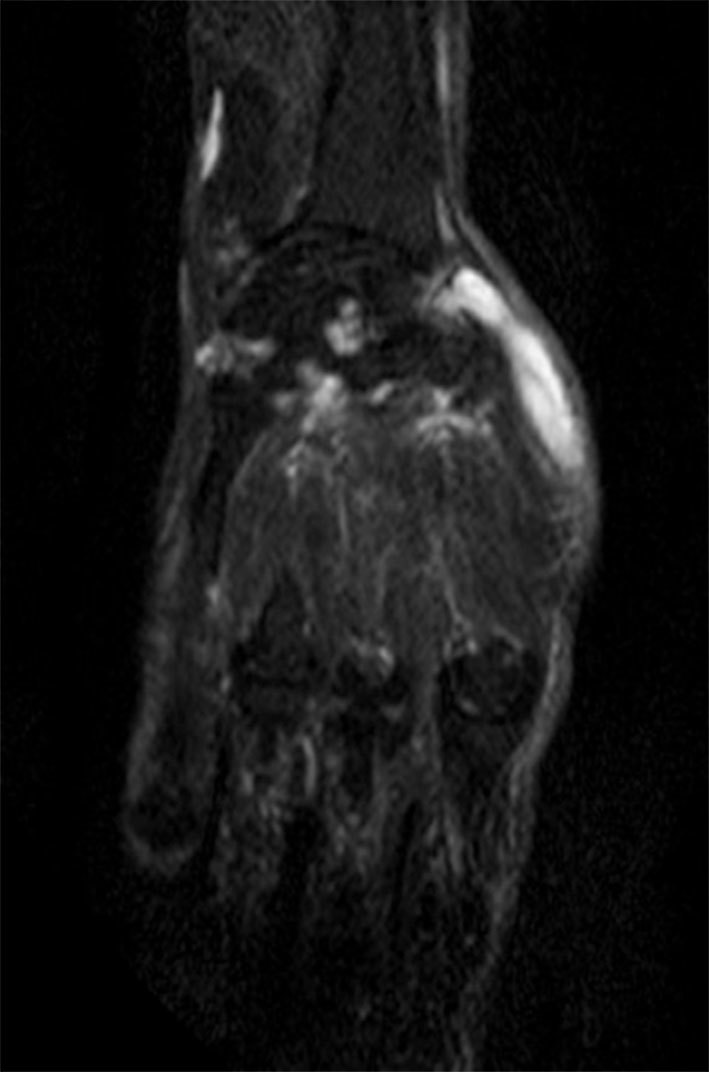
Short tau inversion-recovery MRI showing a cystic lesion involving the lateral aspect of the wrist (along the abductor pollicis longus and the extensor pollicis brevis).

**Figure 7.**
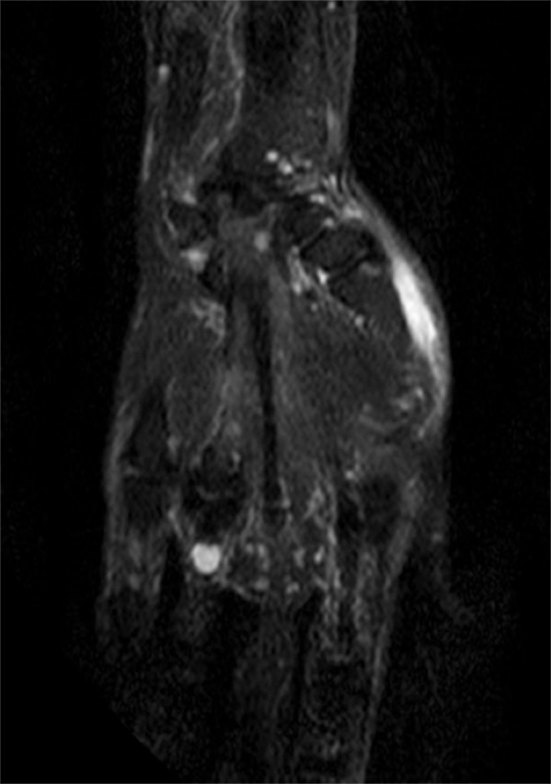
Coronal short tau inversion-recovery MRI of the right hand showing a hyperintense cystic lesion along the flexor tendon at the proximal phalanx of the ring finger.

**Figure 8.**
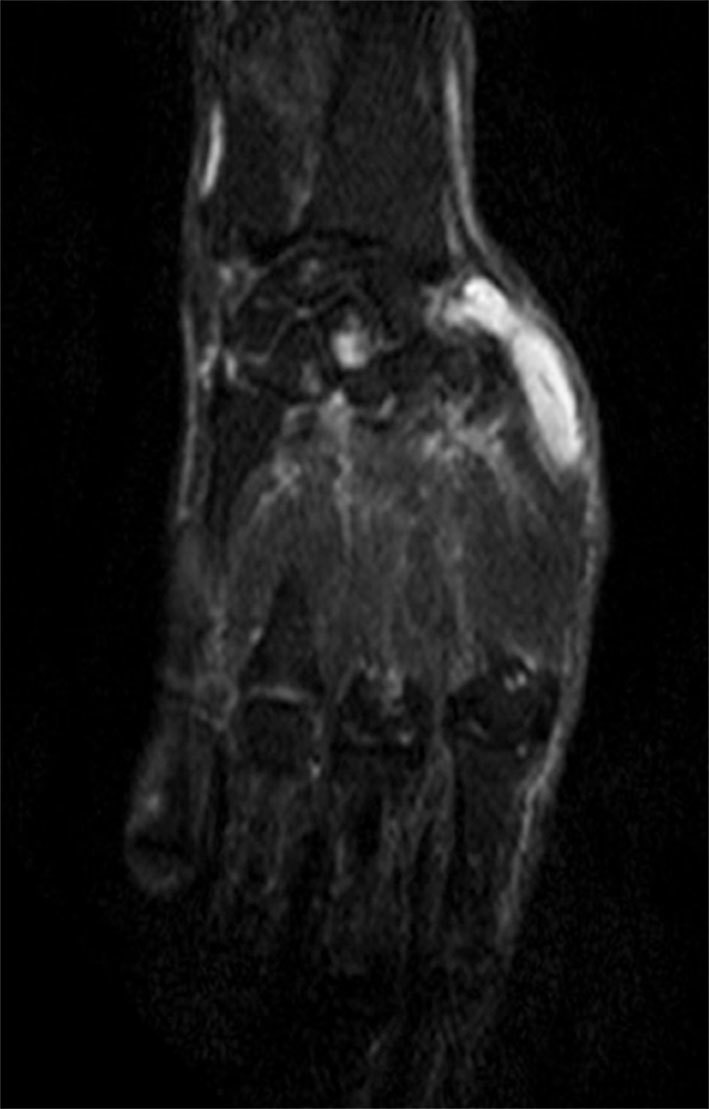
Coronal short tau inversion-recovery MRI of the right hand showing a hyperintense cystic lesion within the capitate and the lunate.

**Figure 9.**
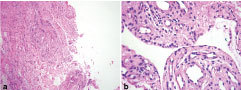
Haematoxylin and eosin-stained slides (a, 10×;b, 40×) showing a cystic lesion lined by a single layer of synoviocytes. The cyst wall is thick and made of dense fibrocollagenous stroma with sparse lymphocytic infiltration. The lumen shows myxoid material, confirming a ganglion cyst.

A 57-year-old female presented with a history of pain and swelling below the left knee, insidious in onset for 4 years with a sudden increase in size over the past 2 months. She also had swelling on the dorsum of the right hand. No significant limitations of movement were noted. No history of injury was given by the patient. An ultrasound of the knee joint showed multiple loculated cystic lesions involving the subcutaneous planes and the popliteal fossa with diffuse internal echoes (Figure 1). It was suggested that she undergo an MRI of the knee for further evaluation, which showed multiloculated cystic lesions that were hypointense on *T*_1_ weighted and hyperintense on *T*_2_ weighted sequences around the knee joint with intra- and extra-articular components (Figures 2 and 3), and subcutaneous extension insinuating between the tendons, muscles and popliteal vessels (Figure 4). A similar small lesion was seen at the non-weight-bearing surface of the lateral condyle of the femur, suggestive of an intraosseous lesion (Figure 5). Hoffa’s fat pad was involved (Figure 5). Underlying degenerative changes were noted in the bilateral knee joints. Similar cystic lesions were found around the right knee joint, right wrist, abductor pollicis longus, extensor pollicis brevis (Figure 6) and overlying the ring finger, in the flexor tendon at the proximal phalanx of ring finger (Figure 7). Intraosseous involvement of the capitate and lunate were noted (Figure 8). With all the above findings and in view of multiple joint involvement, the possibility of cystic ganglionosis or multiple giant ganglion cysts was considered. Aspiration of one of the knee lesions was performed, which yielded a gelatinous material. Fine-needle aspiration cytology showed cyst macrophages in clusters against proteinaceous–mucoid background, without any atypical cells. Also, biopsy of the lesion confirmed the diagnosis of a ganglion cyst (as described in Figure 9a,b). As the lesion was diagnosed to be harmless, the patient was advised conservative management and follow-up. With multi-joint involvement of the ganglion cysts, the term “cystic ganglionosis” was used.

## Discussion

Ganglia are not included in the World Health Organization classification of soft-tissue tumours as they are not true tumours. However, they are very common and should be considered in the differential diagnosis of a soft-tissue mass. Ganglia arise from the joint capsules, bursae, ligaments, tendons and subchondral bone.^1^ They commonly occur in the hands, wrists and feet.

Multiple, extensive ganglion cysts, which we have described in our patient, are extremely rare. The involvement of multiple joints, bilateral knees, intraosseous variety and involvement of Hoffa’s fat pad in a single adult has not been described in the literature. In our extensive literature search, we came across only one case of multiple ganglion cysts reported in an 11-year-old child. As this is probably the first case of an adult with multiple ganglion cysts in a single patient, we have reported this case.

The pathogenesis of ganglion cyst is controversial: two theories have been described, the “myxoid degeneration theory,” which implies that the ganglion cyst arises from a degenerative process and cystic softening of the collagen and the connective tissues secondary to chronic irritation or trauma. On the contrary, “the synovial theory” suggests that the ganglion cyst arises from synovial herniation of the joint capsule or the tendon sheath owing to a defect or traumatic tear.^2^ The ganglia are lined by a capsule composed of flat spindle cells and do not have a synovial lining.^1^ Histologically, ganglion cysts are filled with a highly viscous, gelatinous, proteinaceous material that consists of hyaluronic acid, albumin, globulin and glucosamine.^2^ Ganglion cysts can occur at any age, with girls being affected four times as often as boys. The usual site is the dorsum of the wrist near the radiocarpal joint or over the volar radial aspect of the wrist.^2^ Intraneural ganglion cysts of the peripheral nerves and the temporomandibular joints have also been described.^2^

On MRI, the ganglia are seen as round or ovoid masses with smooth or slightly lobulated surfaces, and are in close proximity to a joint or tendon. They can be uniloculated or multiloculated. They usually appear isointense or slightly hypointense to muscle on *T*_1_ weighted and hyperintense on *T*_2_ weighted MRIs. On contrast MRI, they can demonstrate a thin rim of contrast enhancement, with or without a thin enhancing septae.^3^ Lesions showing hyperintense contents on *T*_1_ weighted images may reflect their higher proteinaceous content. A ganglion can also be seen far away from a joint.^1^ Typical ultrasound features include an anechoic or hypoechoic well-defined mass, oval or round in shape containing debris and lying close to the tendon sheath or the joint capsule. Internal echoes in the ganglion may mimic a solid mass. Some ganglions are compressible.^4,5^ Septations are noted in a ganglion with internal echoes within. Colour and power Doppler may demonstrate mild peripheral vascularity.^5^

Cystic ganglionosis is the presence of multiple ganglion cysts involving multiple joints. The reason and pathogenesis behind the development of multiple ganglion cysts is unclear as this is extremely rare. Further studies need to be performed regarding the pathogenesis and genetic predisposition, if any, behind the multiplicity. The cystic lesions in our patient showed similar ultrasound and MR signal characteristics of ganglion cysts. However, the cysts were so extensive in the bilateral knees that they were seen insinuating between the tendons, muscles and popliteal vessels. Hoffa’s fat pad cysts were noted. Cysts with similar signal intensity were also seen along the tendons of the wrist and the hand. Intraosseous lesions were also seen in the femoral condyle and the capitate and the lunate.

About 50–70% of soft-tissue masses in the wrist region are ganglions.^4^ Although they can occur in various locations, such as the ankles, elbows, hips, shoulders and knees, the wrists and hands are most commonly involved. A rare case of a ganglion cyst arising from Hoffa’s fat pat has been reported.^6^ Another rare case of a lumbar ganglion cyst^7^ and peroneal nerve ganglion cyst^8^ has been reported. Ganglion cysts are usually asymptomatic, but if they are sufficiently large to impinge on the nerves, they can cause weakness, pain, discomfort (as in our patient) with limitation of motion and paraesthesia.^2^

Watchful waiting, non-operative aspiration and surgical removal are the main treatment options. High recurrence rates are noted with cyst aspiration. Surgery is better in terms of lower rates of recurrence, but has a higher incidence of complications.^9^ Before surgery, it is important to confirm the cystic nature of these lesions to determine their relationship to the joint and evaluate the joint for the presence of associated intra-articular disease. A demonstration of the relationship of the cyst to the capsule may be important in differentiating the ganglia from the other lesions, as failure to remove the capsular components of the cyst may lead to recurrence.^10^ A high rate of spontaneous resolution and recurrence after surgery argue against surgical intervention to treat them.

## Conclusions

To our knowledge, no case of multiple ganglion cysts in adults has been reported in the medical literature. Only one case of multiple ganglion cysts has been reported in an 11-year-old child. The multifocal distribution of the ganglion cysts in our patient is unique, also bilateral involvement of the knees has never been reported previously. These may imply some inherent susceptibility of the patient to develop these lesions. The need to screen other joints and look for multifocality is highlighted by this case. We have reported this case because of this rare multiplicity, the unusual presentation involving the bilateral knees and Hoffa’s fat pad, together with multiple intraosseous lesions in a single patient. The importance of the diagnosis is that unnecessary surgical intervention and hospitalization can be avoided, provided the patient’s symptoms are not severe.

## Learning points

1. Ganglion cysts are commonly seen around the joints and tendons, appearing as cystic lesions on imaging, and should be included in the differential diagnosis of any cystic lesion around the joints and tendons.
2. Cystic ganglionosis (multiple ganglion cysts) is a very rare condition, where ganglion cysts involve multiple joints and tendons, as described in our patient. This has been hardly reported in the literature, hence its prevalence and associations are not known and further studies are needed in this regard.
3. Awareness and recognition of this condition by the radiologist is important, as they can guide the clinician on management, which is mainly conservative.
